# Labor and Capital

**Published:** 1882-11

**Authors:** 


### LABOR AND CAPITAL.

The *Christian at Work* has this to say in reference to the present conflict between the workmen and their employers: "We are all familiar enough with the power and beneficence of capital. We know that it is the hen that lays the golden egg, or, to quote the fable of Coriolanus, the stomach that sends nourishment, activity, strength, life, through all the members of the body politic. But labor has also its right to live decently. We would like to see the sewing women who stitch, stitch, stitch, in making shirts for a few cents apiece, combine against the great dealers who think themselves compelled by the laws of trade to offer a human being such pitiful pay, or rather wicked apology for pay. God forbid that the notions of the old world capitalist, who actually rejoices to have his brother man struggling from the cradle to the grave on buttermilk and potatoes, should ever become prevalent in America. This adjustment of labor and capital will never be settled until Christianity teaches great corporations to sing with Robert Burns of the lowliest toiler: 'A man's a man for a' that.'"

It is wrong to speak of the American navy as "the American knavery."

